# Plasma extracellular vesicle microRNAs reflecting the therapeutic effect of the CBP/β-catenin inhibitor PRI-724 in patients with liver cirrhosis

**DOI:** 10.1038/s41598-024-56942-1

**Published:** 2024-03-15

**Authors:** Mayu Yoshida, Juntaro Matsuzaki, Koji Fujita, Masamichi Kimura, Tomohiro Umezu, Noi Tokuda, Tomoko Yamaguchi, Masahiko Kuroda, Takahiro Ochiya, Yoshimasa Saito, Kiminori Kimura

**Affiliations:** 1 Division of Pharmacotherapeutics, Faculty of Pharmacy, Keio University, 1-5-30 Shibakoen, Minato-ku, Tokyo, 105-8512 Japan; 2 Department of Molecular Pathology, Tokyo Medical University, 6-1-1 Shinjuku, Shinjuku-ku, Tokyo, 160-8402 Japan; 3 Department of Hepatology, Tokyo Metropolitan Cancer and Infectious Diseases Center, Komagome Hospital, 3-18-22 Honkomagome, Bunkyo-ku, Tokyo, 113-8677 Japan; 4 Department of Molecular and Cellular Medicine, Institute of Medical Science, Tokyo Medical University, 6-7-1 Nishishinjuku, Shinjuku-ku, Tokyo, 160-0023 Japan

**Keywords:** Extracellular vesicle, miRNA, Antifibrotic therapy, Liver cirrhosis, Bioinformatics, Microarray analysis, RNAi

## Abstract

There is an unmet need for antifibrotic therapies to prevent the progression of liver cirrhosis. Previously, we conducted an exploratory trial to assess the safety and antifibrotic efficacy of PRI-724, a selective CBP/β-catenin inhibitor, in patients with liver cirrhosis. PRI-724 was well tolerated and exerted a potential antifibrotic effect. Here, we investigated whether the profiles of circulating microRNAs packaged in extracellular vesicles (EV-miRNAs) are associated with responses to liver fibrosis treatments. Eighteen patients who received PRI-724 for 12 weeks in a phase 1/2a study were classified as responders (n = 10) or non-responders (n = 8) based on changes in liver stiffness. Plasma samples were obtained before and after PRI-724 administration and the levels of EV-miRNAs were analyzed. Three miRNAs (miR-6510-5p, miR-6772-5p, and miR-4261) were identified as predictors of response or non-response to PRI-724, and the levels of three other miRNAs (miR-939-3p, miR-887-3p, and miR-7112-5p) correlated with the efficacy of treatment. Expression of miR-887-3p was detected in hepatocytes and was decreased significantly in liver tissue following PRI-724 treatment. In addition, transfection of a miR-887-3p mimic activated hepatic stellate cells. Thus, decreases in the miR-887-3p level in blood may reflect recovery from liver fibroses in patients with liver cirrhosis treated with PRI-724, although further validation studies are warranted to confirm this.

## Introduction

Each year, liver disease kills approximately 2 million people worldwide; 1 million of these deaths are related to liver cirrhosis, which is the 11th most common cause of death^1^. Liver fibrosis can progress to cirrhosis as a result of continuous liver damage, and fibrosis is strongly associated with future liver disease-associated morbidity and mortality. Patients with liver cirrhosis require frequent hospitalization, and decompensated cirrhosis leads to an impaired quality of life^1,2^. Furthermore, 1–4% of patients with liver cirrhosis develop hepatocellular carcinoma (HCC)^1^. Given the link between liver fibrosis, cirrhosis, and HCC, there is a need to develop new therapeutic drugs to improve liver fibrosis. The development of antiviral drugs to treat chronic hepatitis is advancing, but an antifibrotic drug does not currently exist.

A link between Wnt signaling and transforming growth factor-β (TGF-β)-mediated fibrosis has been established and is attracting attention as a target for antifibrotic therapy^3,4^. Based on solid evidence demonstrating the antifibrotic efficacy of a selective cyclic AMP-response element binding protein-binding protein (CBP)/β-catenin inhibitor (PRI-724) in mice^5–7^, we recently conducted a single-center, open-label, dose escalation phase 1 trial (PRI-724-1101) to investigate the safety and efficacy of PRI-724 in patients with HCV-induced liver cirrhosis^8^. The 14 patients enrolled in the trial were divided into three groups, each of which received a different dose of PRI-724 (10 mg/m^2^/day, n = 6; 40 mg/m^2^/day, n = 6; 160 mg/m^2^/day, n = 2). A single treatment cycle consisted of continuous intravenous infusion of PRI-724 for 1 week, followed by a 1-week intermission. A marked reduction in fibrosis was observed after six cycles (12 weeks). To reduce the patient burden due to the 1-week continuous treatment, we then conducted a multicenter, open-label, non-randomized, non-placebo-controlled phase 1/2a trial (PRI-724-2101) in patients with HCV- or HBV-induced liver cirrhosis^2^. In phase 1 of the PRI-724-2101 trial, 15 patients were divided into three cohorts and PRI-724 was administered at a dose of 140, 280, or 380 mg/m^2^/4 h, twice a week for 12 cycles. In phase 2a of the trial, a group of 12 patients received PRI-724 at a dose of 280 mg/m^2^/4 h, which was the recommended dose determined in phase 1. Although PRI-724 did not decrease hepatic fibrosis in the analysis of liver biopsy tissues, there were significant improvements in the liver stiffness measure (LSM) and blood-based fibrosis markers, suggesting a potential antifibrotic effect.

microRNAs (miRNAs) are short (17–25 nucleotides), non-coding RNA molecules that regulate gene-expression post-transcriptionally. In the extracellular space, miRNAs are stabilized by attaching to proteins or lipoproteins, or by loading into extracellular vesicles (EVs) such as exosomes^9,10^. Dysfunctional expression of miRNAs is a feature of many pathological processes, including cancer and metabolic, inflammatory, cardiovascular, neurodevelopmental, and autoimmune disorders^11^. In the liver, altered expression of miRNAs is associated with the dysregulation of metabolism, as well as tissue injury, fibrosis, and tumor development^12^. Analyzing circulating miRNAs is beneficial not only for understanding disease-related molecular pathophysiology, but also for identifying new blood-based disease biomarkers for tailored therapy.

In this study, we investigated whether the levels of miRNAs in plasma EVs are associated with therapeutic response to PRI-724 in patients with HCV- or HBV-induced liver cirrhosis. In addition, we evaluated the expression levels of several miRNAs in liver tissue and the relationships between EV-miRNAs and serum cytokines. Through this analysis, we suggest a new functionality of an extracellular miRNA in liver fibrosis.

## Results

### Patient characteristics and responses to PRI-724

**Figure 1:**
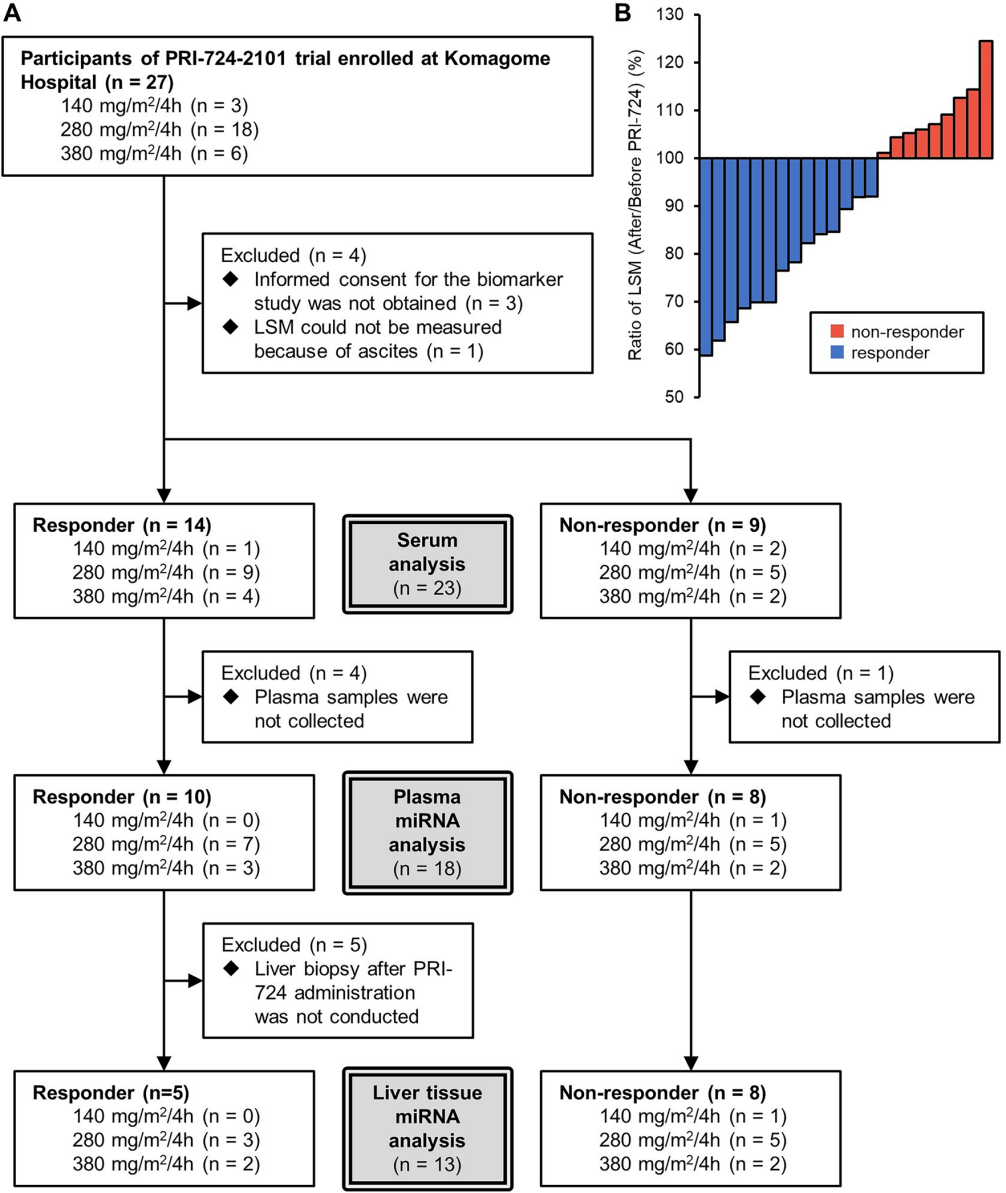
Overview of analyzed samples. (**A**) Informed consent for the biomarker study was obtained before sample collection and was obtained separately from that for the PRI-274-2101 trial. The numbers of collected samples for serum, plasma miRNA, and liver tissue miRNA analyses differed. (**B**) The ratio of LSM after/before PRI-724 administration in the 23 participants. Values lower than 100% indicate an improvement in LSM and those higher than 100% indicate aggravation of LSM.

Between July 2018 and July 2021, 27 patients with liver cirrhosis were registered in the PRI-724-2101 trial. Among these patients, all of those with HCV-induced cirrhosis achieved a sustained virologic response, whereas all of those with HBV-induced cirrhosis were taking nucleic acid synthesis inhibitors and their viral load was below detection^2^. Of these, 3, 18, and 6 patients received PRI-724 at 140, 280, and 380 mg/m^2^/4 h, respectively (Fig. 1). Among them, 24 agreed to be included in the biomarker study. One patient was excluded because he/she had too much ascites before the PRI-724 administration; therefore, LSM could not be measured. Serum samples were collected from 23 patients and serum cytokine array data were obtained. A plasma EV-miRNA microarray analysis was also performed for 18 patients and a liver tissue miRNA microarray analysis was performed for 13 patients. The reason for the decrease in the number of eligible patients was that consent for sample acquisition was not obtained in some cases.

Most of the 23 patients included in the study were male and the average age was 62.1 years. Based on a decrease in LSM after PRI-724 administration, 14 patients were classified as responders, and based on an increase in LSM after administration, nine patients were classified as non-responders (Fig. 1). The characteristics of the participants (dose of PRI-724, hepatitis virus infection, sex, and age) did not differ significantly between the responders and non-responders (Table 1). The baseline LSM and baseline fibrosis-4 (FIB-4) index were both significantly higher for the responders than for the non-responders (*P* = 0.013 and 0.006, respectively) (Table 1).

**Table 1:** Participant characteristics.

		Responder (n = 14)	Non-responder (n = 9)	*P*-value
Age [years]	Average (range)	61.3 (51–70)	63.3 (50–72)	0.50^a^
Sex	n (%)			0.52^b^
Men		11 (79)	8 (89)	
Women		3 (21)	1 (11)	
Dose [mg/m^2^/4 h]	n (%)			0.42^b^
140		1 (7)	2 (22)	
280		9 (64)	5 (56)	
380		4 (29)	2 (22)	
Hepatitis virus	n (%)			0.94^b^
HBV (HBV-DNA negative)		6 (43)	4 (44)	
HCV (SVR)		8 (57)	5 (56)	
Child–Pugh score	n (%)			0.31^b^
A		8 (57)	7 (78)	
B		6 (43)	2 (22)	
Baseline LSM	Average (range)	22.7 (8.8–35.3)	14.2 (5.3–26.3)	**0.013**^a^
Baseline ELF	Average (range)	11.0 (9.1–13.5)	10.5 (9.2–13.7)	0.34^a^
Baseline FIB-4	Average (range)	5.7 (1.1–10.1)	3.0 (1.7–5.1)	**0.006**^a^
Baseline PIIIP	Average (range)	14.5 (6.3–29.1)	15.4 (5.7–57.9)	0.88^a^

Bold letters indicate a statistically significant difference between the responder and non-responder groups.

HBV, hepatitis B virus; HCV, hepatitis C virus; LSM, liver stiffness measure; ELF, enhanced liver fibrosis; FIB-4, fibrosis-4 index; PIIIP, type III procollagen-N-peptide.

^a^unpaired *t*-test.

^b^χ^2^ test.

### Identification of plasma EV-miRNAs that predict the efficacy of PRI-724

**Figure 2:**
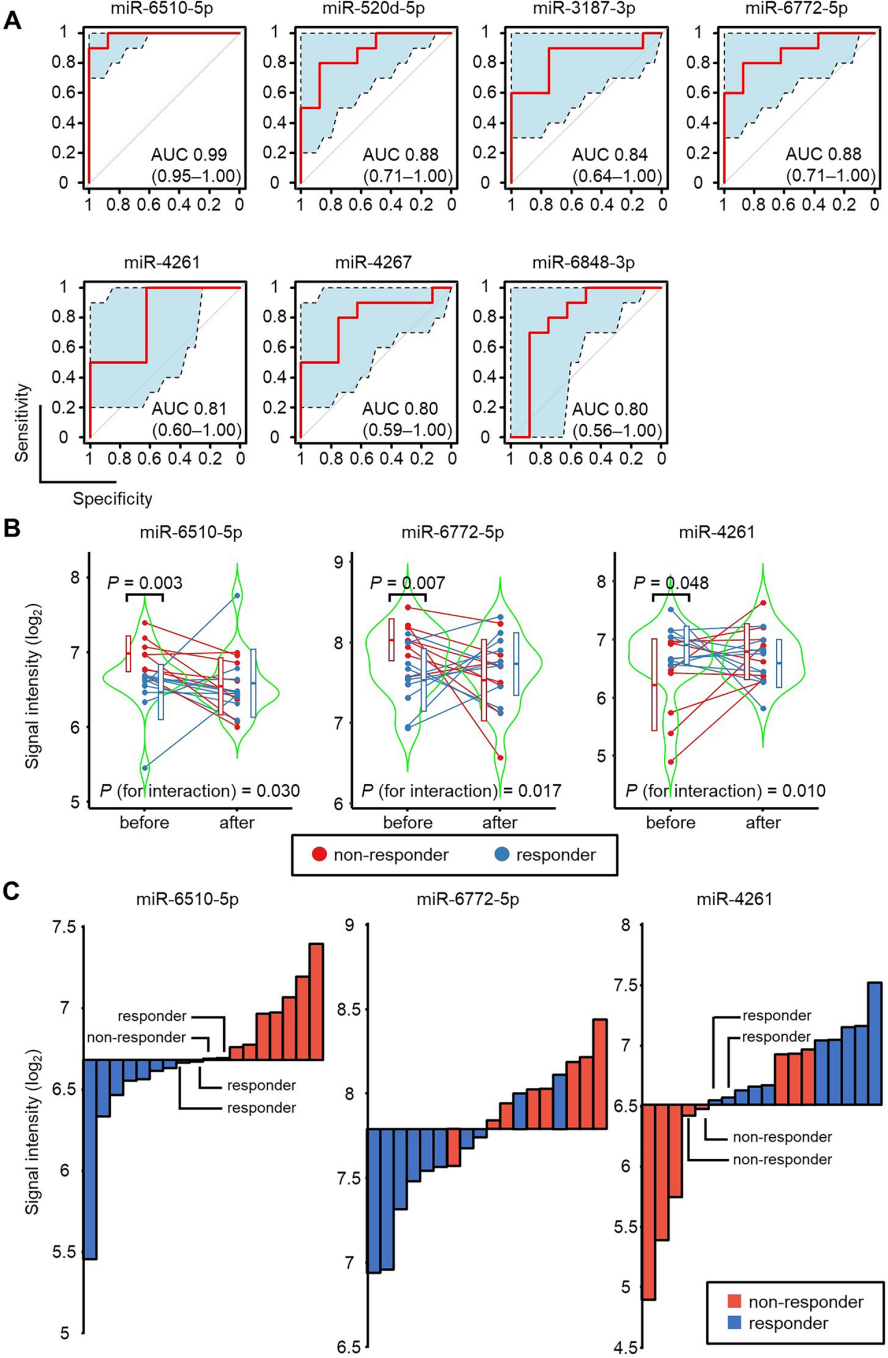
Selection of plasma EV-miRNAs that can be used to predict the therapeutic effect of PRI-724. (**A**) ROC analyses of miR-6510-5p, miR-520d-5p, miR-3187-3p, miR-6772-5p, miR-4261, miR-4267, and miR-6848-3p. The AUC is shown for each miRNA. Blue shaded areas and dashed lines indicate 95% confidence intervals for the ROC curves. (**B**) Violin plots of the signal intensities of miR-6510-5p, miR-6772-5p, and miR-4261 in plasma EVs from responders (blue) and non-responders (red) before and after PRI-724 administration. Unpaired *t*-tests were used to analyze statistically significant differences between the responders and non-responders before PRI-724 administration. For each miRNA, the statistical significance of the interaction was calculated to determine whether the effect of PRI-724 on the miRNA level differs between responders and non-responders. (**C**) The relationship between the baseline signal intensities of miR-6510-5p, miR-6772-5p, and miR-4261 and the patients’ responses to PRI-724 (responder vs. non-responder).

To identify plasma EV-miRNAs that could be used to predict a patient’s response to PRI-724, we searched for EV-miRNAs with baseline levels that differed between responders and non-responders. Of the 2632 miRNAs included in the analysis, 849 miRNAs had a signal intensity higher than the predefined threshold and were selected for further analysis. Seven of these miRNAs (miR-6510-5p, miR-520d-5p, miR-3187-3p, miR-6772-5p, miR-4261, miR-4267, and miR-6848-3p) achieved a cross-validation score  > 0.75 and AUC  > 0.8 ([cross-validation score, AUC]: miR-6510-5p [0.83, 0.99]; miR-520d-5p [0.83, 0.88]; miR-3187-3p [0.83, 0.84]; miR-6772-5p [0.78, 0.88]; miR-4261 [0.78, 0.81]; miR-4267 [0.78, 0.80]; miR-6848-3p [0.78, 0.80]) (Table 2, Fig. 2A). Among these seven miRNAs, significant interaction effects between responders and non-responders were observed for miR-6510-5p (*P* = 0.030), miR-6772-5p (*P* = 0.017), and miR-4261 (*P* = 0.010) (Fig. 2B, Supplemental Fig. 1).

**Table 2:** List of miRNAs that predict response to PRI-724.

Rank	miRNA	AUC	Cross-validation score
1	**miR-6510-5p**	0.99	0.83
1	**miR-520d-5p**	0.88	0.83
1	**miR-3187-3p**	0.84	0.83
4	**miR-6772-5p**	0.88	0.78
4	**miR-4261**	0.81	0.78
4	**miR-4267**	0.80	0.78
4	**miR-6848-3p**	0.80	0.78
4	miR-4323	0.79	0.78
4	miR-4761-3p	0.79	0.78
4	miR-4462	0.78	0.78
4	miR-6792-3p	0.69	0.78
4	miR-4523	0.68	0.78
4	miR-4731-5p	0.66	0.78

Bold letters indicate miRNAs with AUC  > 0.8 and a cross-validation score  > 0.75.

The percentage of patients with a low baseline miR-6510-5p level who were classified as responders was significantly higher than the percentage of patients with a high miR-6510-5p level who were classified as responders (100% [9/9] vs. 11% [1/9]; *P* = 0.0001; Fig. 2C). Similarly, the miR-6772-5p-low group had a significantly higher rate of responders than the miR-6772-5p-high group (89% [8/9] vs. 22% [2/9]; *P* = 0.004; Fig. 2C). By contrast, the miR-4261-high group had a significantly higher rate of responders than the miR-4261-low group (77% [10/13] vs. 0% [0/5]; *P* = 0.003; Fig. 2C).

### Identification of plasma EV-miRNAs that monitor the efficacy of PRI-724

**Figure 3:**
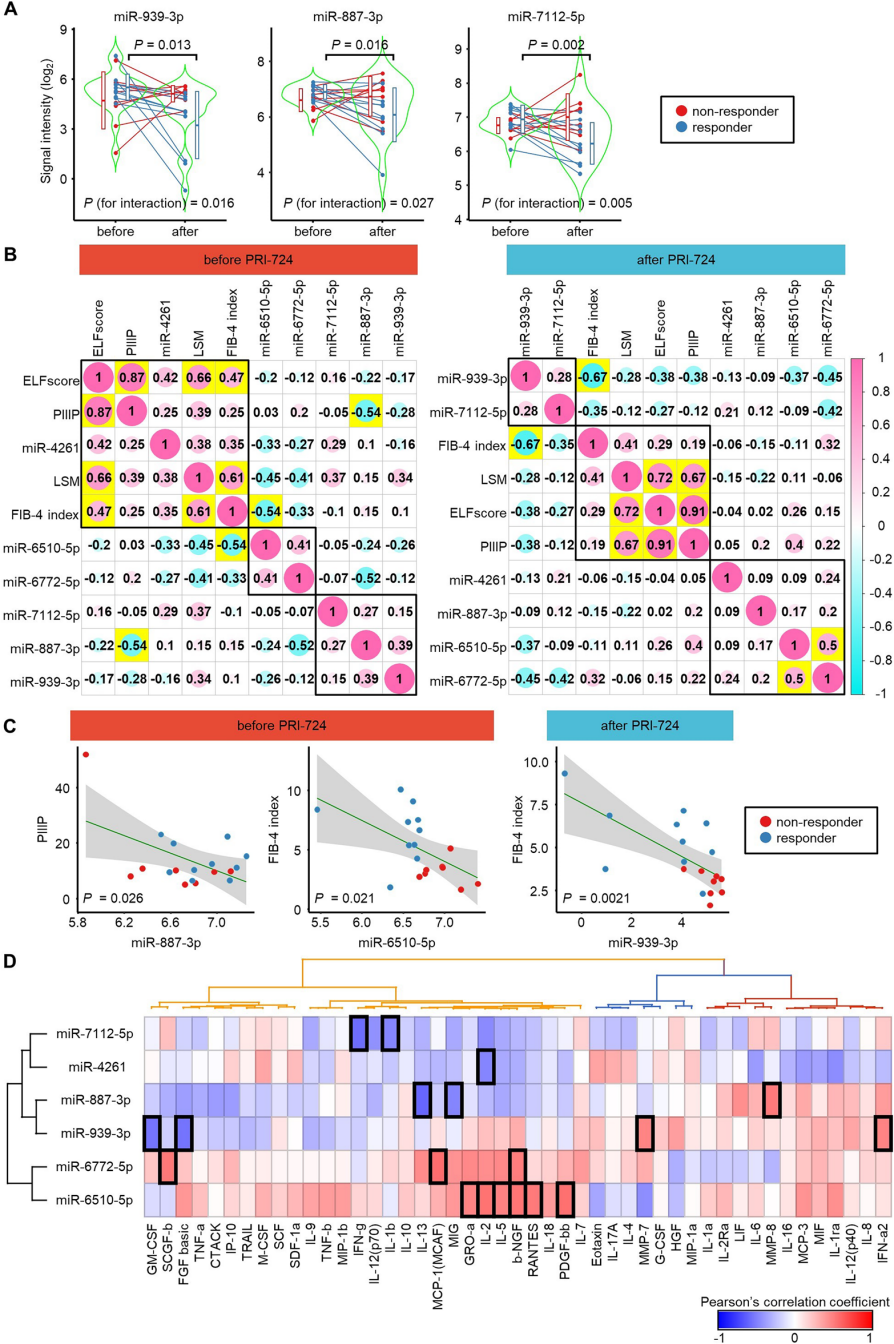
Plasma EV-miRNAs that can be used to monitor the therapeutic effect of PRI-724, and correlations between the expression levels of miRNAs and fibrosis markers. (**A**) Violin plots of the signal intensities of miR-939-3p, miR-887-3p, and miR-7112-5p in serum EVs from responders and non-responders before and after PRI-724 administration. Paired *t*-tests were used to analyze statistically significant differences between the baseline and after PRI-724 administration for the responders. For each miRNA, the statistical significance of the interaction was calculated to determine whether the effect of PRI-724 on the miRNA level differs between responders and non-responders. (**B**) Correlations between the fibrosis markers and the expression levels of the six plasma EV-miRNAs for which significant interaction effects were analyzed before (left panel) and after (right panel) treatment with PRI-724 (n = 18). Numbers indicate Pearson's correlation coefficients. Yellow boxes indicate statistical significance (*P* < 0.05). (**C**) Correlation plots between significantly correlated pairs of fibrosis markers and the expression levels of plasma EV-miRNAs are shown. *P* values were calculated by Pearson's correlation analysis. (**D**) Correlations between the baseline serum levels of cytokines and the baseline expression levels of the six plasma EV-miRNAs for which significant interaction effects were observed (n = 18). Colors indicate Pearson's correlation coefficients. Black boxes indicate statistical significance (*P* < 0.05).

To identify plasma EV-miRNAs that could be used to monitor responses to PRI-724, we searched for EV-miRNAs whose levels differed in responders before and after treatment. Eight EV-miRNAs (miR-6795-3p, miR-939-3p, miR-887-3p, miR-4687-5p, miR-181d-3p, miR-7112-5p, miR-765, and miR-4292) achieved a cross-validation score > 0.75 and AUC > 0.8 ([cross-validation score, AUC]: miR-6795-3p [0.85,0.95]; miR-939-3p [0.85,0.93]; miR-887-3p [0.85,0.81]; miR-4687-5p [0.85,0.81]; miR-181d-3p [0.75,0.84]; miR-7112-5p [0.75,0.84]; miR-765 [0.75,0.80]; and miR-4292 [0.75,0.80]) (Table 3). Among these eight miRNAs, significant interaction effects between responders and non-responders were observed for miR-939-3p (*P* = 0.016), miR-887-3p (*P* = 0.027), and miR-7112-5p (*P* = 0.005) (Fig. 3A, Supplemental Fig. 2). These trends did not differ between the administered dosages of PRI-724 (140, 280, or 380 mg/m^2^/4 h) (Supplemental Fig. 3).

**Table 3:** List of miRNAs that monitor response to PRI-724 during treatment.

Rank	miRNA	AUC	Cross-validation score
1	**miR-6795-3p**	0.95	0.85
1	**miR-939-3p**	0.93	0.85
1	**miR-887-3p**	0.81	0.85
1	**miR-4687-5p**	0.81	0.85
5	miR-625-3p	0.78	0.80
6	**miR-181d-3p**	0.84	0.75
6	**miR-7112-5p**	0.84	0.75
6	**miR-765**	0.80	0.75
6	**miR-4292**	0.80	0.75
6	miR-5008-3p	0.79	0.75
6	miR-7106-5p	0.78	0.75
6	miR-193b-5p	0.76	0.75
6	miR-1266-5p	0.76	0.75
6	miR-6792-3p	0.76	0.75
6	miR-7843-5p	0.75	0.75
6	miR-6780b-5p	0.74	0.75
6	miR-6776	0.73	0.75
6	miR-6858-3p	0.73	0.75
6	miR-3173-3p	0.65	0.75
6	miR-4640-5p	0.65	0.75

Bold letters indicate miRNAs with AUC  > 0.8 and a cross-validation score  > 0.75.

### Correlations between the levels of plasma EV-miRNAs and levels of fibrosis markers

Next, we examined the relationship between the levels of fibrosis markers and the levels of the six plasma EV-miRNAs for which significant interaction effects were observed, including the three miRNAs that predicted the therapeutic effect of PRI-724 (miR-6510-5p, miR-6772-5p, miR-4261) and the three miRNAs that monitored its therapeutic effect (miR-939-3p, miR-887-3p, miR-7112-5p) in all 18 patients subjected to plasma EV-miRNA analysis (Fig. 3B). Prior to PRI-724 administration, there were significant correlations between the miR-887-3p level and the type III procollagen-N-peptide (PIIIP) level (R = − 0.54, *P* = 0.026) and between the miR-6510-5p level and the FIB-4 index (R = − 0.54, *P* = 0.021) (Fig. 3C). After PRI-724 administration, there was a significant correlation between miR-939-3p level and the FIB-4 index (R = − 0.67, *P* = 0.0021) (Fig. 3C).

### Correlations between the levels of plasma EV-miRNAs and serum cytokine levels

Analysis of the baseline levels of serum cytokines in responders and non-responders revealed that no cytokines were associated significantly with the treatment response (Supplemental Table 1). Subsequently, we examined the relationship between the serum levels of cytokines and the levels of the previously identified six plasma EV-miRNAs (miR-6510-5p, miR-6772-5p, miR-4261, miR-939-3p, miR-887-3p, and miR-7112-5p) in plasma EV-miRNA analysis. Prior to PRI-724 administration, there were significant correlations between miR-7112-5p and INF-g (interferon-gamma; R = − 0.571, *P* = 0.013) or IL-1b (interleukin-1 beta; R = − 0.475, *P* = 0.046); between miR-4261 and IL-2 (R = − 0.475, *P* = 0.047); between miR-887-3p and IL-13 (R = − 0.592, *P* = 0.010), MIG (monokine induced by gamma; R = − 0.477, *P* = 0.045), or MMP-8 (matrix metalloproteinase 8; R = 0.521, *P* = 0.027); between miR-939-3p and GM-CSF (granulocyte–macrophage colony-stimulating factor; R = − 0.543, *P* = 0.020), FGF basic (fibroblast growth factor basic; R = − 0.541, *P* = 0.020), MMP-7 (R = 0.477, *P* = 0.045), or IFN-a2 (R = 0.480, *P* = 0.044); between miR-6772-5p and SCGF-b (stem cell growth factor beta; R = 0.522, *P* = 0.026), MCP-1 (monocyte chemotactic protein 1; R = 0.566, *P* = 0.014), or b-NGF (beta nerve growth factor; R = 0.510, *P* = 0.031); and between miR-6510-5p and GRO-a (growth-related oncogene alpha; R = 0.479, *P* = 0.044), IL-2 (R = 0.546, *P* = 0.019), IL-5 (R = 0.493, *P* = 0.038), b-NGF (R = 0.544, *P* = 0.020), RANTES (regulated on activation, normal T cell expressed and secreted; R = 0.520, *P* = 0.027), or PDGF-bb (platelet derived growth factor BB; R = 0.553, *P* = 0.017) (Fig. 3D).

### Hepatic expression of the identified miRNAs

**Figure 4:**
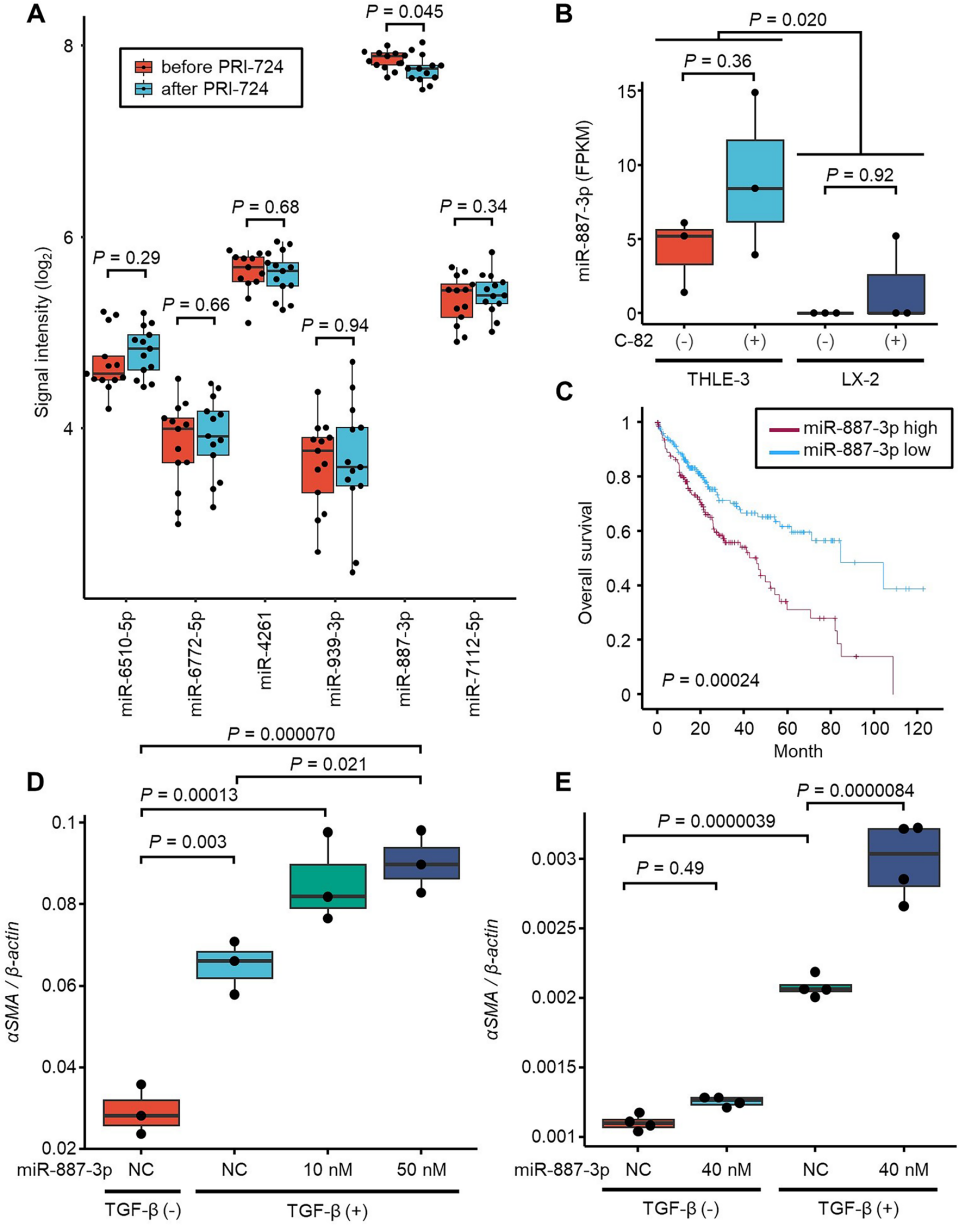
Expression levels of selected miRNAs in the liver and analysis of the function of miR-887-3p. (**A**) Signal intensities in liver tissue of the six miRNAs for which significant interaction effects were observed (n = 13).* P* values were calculated by paired *t*-tests. (**B**) Comparison of the expression of miR-887-3p in immortalized hepatocyte cells (THLE-3) and immortalized hepatic stellate cells (LX-2) with and without exposure to C-82, the active form of PRI-724. Experiments were repeated three times. *P* values were calculated by one-way ANOVA with Tukey’s post-hoc analysis. (**C**) Kaplan–Meier plot of miR-887-3p expression in HCC using a public dataset. *P* values were calculated by log-rank test. (**D**) The expression level of the *αSMA* mRNA expression in HHSteC transfected with a miR-887-3p mimic or negative control scramble RNA and treated with TGF-β. Experiments were repeated three times. *P* values were calculated by one-way ANOVA with Tukey’s post-hoc analysis. (**E**) The expression level of *αSMA* mRNA in LX-2 cells transfected with a miR-887-3p mimic or negative control scramble RNA and treated with TGF-β. Experiments were repeated four times.* P* value was calculated by one-way ANOVA with Tukey’s post-hoc analysis.

Next, we examined the hepatic expression levels of the six miRNAs for which significant interaction effects were observed in all 13 patients subjected to liver tissue miRNA analysis. Only miR-887-3p had a normalized signal intensity  > 2^6^ and was expressed at significantly different levels before and after PRI-724 administration (*P* = 0.045) (Fig. 4A).

To identify the major source of miR-887-2p in the liver, we conducted ISH analysis for miR-887-3p in the liver tissues of the participants in this trial. The results suggested that miR-887-3p is expressed in hepatocytes, as observed previously for the hepatocyte-specific miRNA, miR-122-3p^13^ (Supplemental Fig. 4). To confirm the expression of miR-887-3p in hepatocytes, we checked for changes in the miRNA profiles in immortalized hepatocyte and hepatic stellate cell lines (THLE-3 and LX-2, respectively) after exposure to C-82, the active form of PRI-724, using miRNA-seq analysis. miR-887-3p expression was detected in THLE-3 cells but its level was unaffected by exposure to C-82 (Fig. 4B), whereas its expression was hardly detectable in LX-2 cells. This result confirmed that hepatocytes are a major source of miR-887-3p in the liver, as suggested by the results of in situ hybridization; however, conversely, they showed that miR-887-3p expression was unaffected by C-82. Slight elevation of miR-887-3p by C-82, although not statistically significant, could be because CBP/β-catenin signaling is normal in THLE-3 cells unlike in the hepatocytes of the cirrhotic liver.

Importantly, higher expression of miR-887-3p in HCC tissue was associated with worse prognosis according to the TCGA-LIHC dataset^14^ (Fig. 4C). Cox regression analysis revealed that the tissue level of miR-887-3p was an independent prognostic factor for patients with HCC (Table 4). These results suggested that decreasing the level of miR-887-3p might have important cytoprotective effects in the liver.

**Table 4:** Cox regression analysis of overall survival.

	Univariable analysis		Multivariable analysis	
	HR	(95% CI)	HR	(95% CI)
miR-887-3p (per 2 times)	**1.25**	(**1.06–1.47**)	**1.22**	(**1.03–1.45**)
Stage				
I	Ref		Ref	
II	1.47	(0.90–2.40)	1.47	(0.87–2.34)
III	**2.76**	(**1.80–4.23**)	**2.74**	(**1.79–4.20**)
IV	**5.98**	(**1.84–19.4**)	**5.28**	(**1.59–17.5**)
Unknown	**2.90**	(**1.58–5.35**)	**2.61**	(**1.39–4.88**)
Sex				
Women	ref		ref	
Men	1.23	(0.85–1.74)	1.01	(0.70–1.47)
Age (per 10 year)	1.14	(0.99–1.30)	1.10	(0.96–1.27)

Bold letters indicate statistical significance.

HR, hazard ratio; CI, confidence interval.

### miRNA levels changed after hepatocyte exposure to C-82

We also explored whether liver tissue miRNA levels in the participants were altered by administration of PRI-724 and whether these alterations were consistent with those observed after exposure of THLE-3 cells to C-82. We identified five miRNAs (miR-671-5p, miR-532-3p, miR-1908-5p, miR-1275, and miR-744-5p) whose levels were influenced by PRI-724 exposure (Supplemental Fig. 5). Notably, the patterns of changes in the levels of these miRNAs in liver tissue before and after administration of PRI-724 were quite similar between responders and non-responders. This suggests that the strength of the pharmacological effect of PRI-724 in liver tissue cannot explain the difference between responders and non-responders.

**Figure 5:**
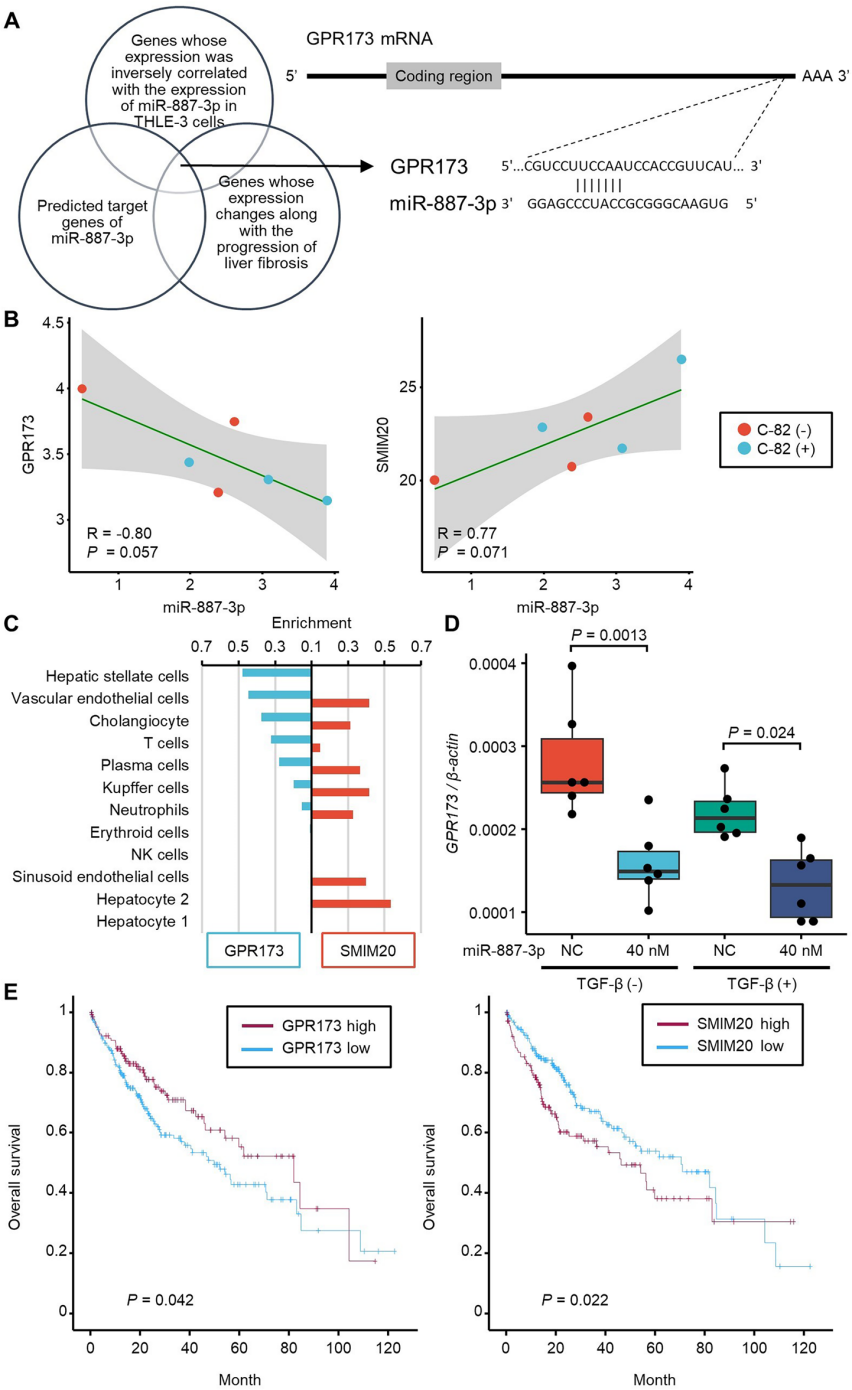
Predicted target gene of miR-887-3p. (**A**) Strategy for identifying predicted target genes of miR-887-3p. A predicted target site on the target gene mRNA sequence is also shown. (**B**) Correlation plots between miR-887-3p and GPR173 expression (left panel) and between miR-887-3p and SMIM20 expression in THLE-3 cells based on the mRNA-seq and miRNA-seq data obtained following exposure to C-82 performed three times in each condition. *P* values were calculated by Pearson's correlation analysis. (**C**) GPR173 and SMIM20 expression in each cell type in the liver. (**D**) The expression level of the *GPR173* mRNA in LX-2 cells transfected with a miR-887-3p mimic or negative control scramble RNA and treated with TGF-β. Experiments were repeated six times.* P* values were calculated by one-way ANOVA with Tukey’s post-hoc analysis. (**E**) Kaplan–Meier plots of GPR173 and SMIM20 mRNA expressions in HCC using a public dataset.* P* values were calculated by log-rank tests.

### Overexpression of miR-887-3p increases the expression of αSMA in hepatic stellate cells

A key mechanism driving the development of liver fibrosis is the activation of hepatic stellate cells, which produce extracellular matrix when activated^15^. Therefore, we assessed the expression of *αSMA*, a marker gene of the activation of hepatic stellate cells, after the transfection with an miR-887-3p mimic. A qRT-PCR analysis was performed to examine the effect of overexpression of miR-887-3p on *αSMA* mRNA expression in HHSteC (Fig. 4D) and LX-2 (Fig. 4E). In the presence of TGF-β, miR-887-3p significantly increased the expression level of the *αSMA* mRNA, suggesting that miR-887-3p may exacerbate TGF-β-mediated liver fibrosis. As *αSMA* mRNA does not have a binding site of miR-887-3p according to sequence-based target prediction, miR-887-3p indirectly regulate the expression of αSMA.

### Target gene prediction of miR-887-3p

To further understand the function of miR-887-3p in the liver, we explored the target genes of miR-887-3p by combining the results of mRNA-seq and miRNA-seq in THLE-3 cells, a target prediction algorithm (TargetScanHuman rel. 8.0, https://www.targetscan.org/vert_80/)^16^, and a transcriptome analysis of human fibrotic liver tissues reported previously^17^ (Fig. 5A). Through this approach, we identified GPR173 (G protein-coupled receptor 173) as a potential target gene of miR-887-3p. GPR173 and its ligand, PHX (phoenixin), are known to play diverse roles in the hypothalamic control of reproduction, appetite modulation, and regulation of energy metabolism and inflammation^18^. PHX is a class of pleiotropic neuropeptide that is cleaved from the C-terminal region of its precursor protein SMIM20 (small integral membrane protein 20). The mRNA expression of GPR173 was inversely correlated with the expression of miR-887-3p, whereas the mRNA expression of SMIM20 was positively correlated with the expression of miR-887-3p in THLE-3 cells (Fig. 5B). GPR173 and SMIM20 mRNA expression did not change after exposure to C-82. According to the GTEx Consorium atlas^19^, GPR173 is mostly expressed in hepatic stellate cells, whereas SMIM20 is mostly expressed in hepatocytes in the liver (Fig. 5C). We observed that overexpression of miR-887-3p significantly decreased GPR173 mRNA expression in LX-2 cells (Fig. 5D) irrespective of exposure to TGF-β. Higher expression of GPR173 in HCC tissue was associated with a better prognosis, whereas higher expression of SMIM20 in HCC tissue was associated with a poorer prognosis^20^ (Fig. 5E). These results suggested that PHX released from hepatocytes could bind to its receptor GPR173 on hepatic stellate cells and play a cytoprotective role in the liver, whereas miR-887-3p released from hepatocytes via EVs would inhibit the expression of GPR173 and disturb the PHX-GPR173 signaling pathway.

## Discussion

In order to use PRI-724 effectively in clinical practice, a clear rationale must be established for its administration by determining which patients are likely to benefit from PRI-724 treatment. This study goes some way toward fulfilling this aim by suggesting that such patients could be selected by analyzing their miRNA expression patterns before administration of PRI-724. We found that patients with low miR-6510-5p levels, low miR-6772-5p levels, or high miR-4261 levels in plasma EVs before treatment were more likely to show an improvement in liver fibrosis after PRI-724 administration. Hence, these miRNAs could be used as biomarkers to predict the therapeutic efficacy of PRI-724. Each miRNA was able to discriminate responders from non-responders with high sensitivity and specificity. Notably, no similar biomarkers were identified in the cytokine array. The baseline LSM (AUC, 0.76 [95% confidence interval (CI), 0.56–0.97]) and FIB-4 index (AUC, 0.75 [95% CI, 0.53–0.96]) also differed significantly between responders and non-responders (LSM: 22.7 and 14.2, respectively, *P* = 0.013; FIB-4 index, 5.7 and 3.0, respectively, *P* = 0.006). These results suggest that patients with more progressive liver fibrosis are more likely to benefit from PRI-724 therapy. As the levels of miR-6510-5p, miR-6772-5p, and miR-4261 were not correlated significantly with the severity of fibrosis, examining the degree of fibrosis and measuring EV-miRNA levels could be used orthogonally to improve the estimation of treatment responses.

Minimally or non-invasive methods for monitoring responses during treatment can reduce patient distress caused by repeated liver needle biopsy. Here, we identified miR-939-3p, miR-887-3p, and miR-7112-5p as biomarkers for monitoring patient responses to PRI-724. For all three miRNAs, a decrease in the expression level reflected the therapeutic efficacy of PRI-724. We focused on miR-887-3p because it is expressed highly in the liver. An ISH analysis of human liver tissues confirmed that miR-887-3p is expressed in hepatocytes. The tissue expression levels of miR-887-3p were decreased significantly after PRI-724 administration. In addition, an in vitro experiment suggested that miR-887-3p could activate hepatic stellate cells. By exploring the potential target genes of miR-887-3p, we found that the PHX-GPR173 pathway could have antifibrotic properties. In fact, a previous study reported that administration of PHX improved steatohepatitis induced by high fat diet in mice^21^. Overall, these findings suggest that miR-887-3p may be a profibrotic miRNA released from hepatocytes.

Plasma EV-miR-887-3p levels were positively correlated with serum MMP-8 levels, but were negatively correlated with serum IL-13 and MIG (CXCL9) levels. MMP-8 is a neutrophil collagenase that acts on type I collagen as its preferential substrate. In addition, MMP-8 can also degrade IL-13 and MIG^22–24^. In a previous study, circulating miR-887-3p levels were elevated in patients with acute respiratory distress syndrome (ARDS), and miR-887-3p increased the endothelial release of chemokines and facilitated trans-endothelial neutrophil migration^25^. Considering that neutrophils have a dual role in the liver, whereby they induce hepatocyte injury but promote liver regeneration by secreting MMPs^26^, it is possible that miR-887-3p could play an important role in liver remodeling.

The serum level of miR-939-5p, which is transcribed with miR-939-3p, is elevated in cirrhotic patients with HCC^27^. In addition, miR-939-5p can promote cytokine-induced translational blockade of the human inducible nitric-oxide synthase (iNOS) protein in HCC^28^. As iNOS can cause liver injury^29^, miR-939-5p would be expected to have an anti-inflammatory function by suppressing iNOS expression. Although high expression of miR-939-3p in liver tissue was not observed in this study, further studies of the association between miR-939-3p/5p and liver diseases are warranted.

The limitations of the current study are the small number of participants and the absence of a placebo control group. Since the present clinical trial was a phase 1/2a study and a randomized controlled trial to verify the efficacy of PRI-724 is still in the preparation stage, there is no solid evidence so far that PRI-724 improves liver fibrosis. Also, it is unclear whether the identified miRNAs reflect an improvement in liver fibrosis regardless of the type of antifibrotic treatment. To address this point, it is important to understand the upstream molecular regulatory mechanisms of miR-887-3p in hepatocytes. Further investigations are required to overcome these limitations.

In conclusion, we found that miR-6510-5p, miR-6772-5p, and miR-4261 in plasma EVs could be useful for predicting patient responses to PRI-724, whereas miR-939-3p, miR-887-3p, and miR-7112-5p could be useful for monitoring the therapeutic effect of PRI-724. Among these miRNAs, only miR-887-3p was secreted from hepatocytes and may have pro-fibrotic functions by targeting GPR173 in hepatic stellate cells. Further studies to elucidate the mechanisms underlying these associations will provide new insights into the improvement of liver fibrosis by PRI-724.

## Methods

### Study population

Participants in this study were the patients with HCV- or HBV-induced liver cirrhosis who received PRI-724 (PubChem database CID: 71509318) in the PRI-724-2101 trial (multicenter, open-label, non-randomized, non-placebo-controlled phase 1/2a study) between July 2018 and July 2021. Patients were included in the PRI-724-2101 trial if they were aged 20–74 years and had been diagnosed with HCV- or HBV-induced liver cirrhosis according to condition I or II (I: serum HCV-RNA positive or HCV-antibody positive [irrespective of viral load or treatment]; II: serum HBV-DNA positive or HBs-antibody positive [irrespective of viral load or treatment]) as well as condition III (III: diagnosis of liver cirrhosis confirmed by a liver biopsy performed during the screening period [modified HAI fibrosis score of 5 or 6 or Metavir score of F4]). Participants who received a 12-week administration of PRI-724 at Tokyo Metropolitan Komagome Hospital were enrolled in the current study. Patient characteristics, liver biopsy samples, and blood samples were collected before and within 2 weeks after the last administration. To measure the therapeutic efficacy of PRI-724, LSM was evaluated using vibration-controlled transient elastography (FibroScan; Echosens, Waltham, MA, USA) before and after the administration. Participants with LSM lower than 100% (after vs. before PRI-724 administration) were defined as responders, whereas those with ratios of 100% or higher were defined as non-responders. The detailed data of LSM obtained in this trial had been published^2^.

### Plasma small EV isolation

Peripheral venous blood was collected in EDTA-2Na tubes before and after PRI-724 administration. The samples were centrifuged at 3500 rpm (about 2000 xg) for 20 min at 4 °C, and the plasma supernatant was stored at − 80 °C until use. Adulterants in the plasma were removed by centrifuging at 10,000 g for 30 min at 4 °C. Subsequently, EVs were isolated from 1 mL of plasma via the TIM4-affinity method^30^, using the MagCapture Exosome Isolation Kit PS (Wako, Osaka, Japan), according to the manufacturer's instruction. The quality of the isolated EVs was checked using the NanoSight NS300 instrument (Malvern Panalytical, Malvern, UK).

### miRNA microarray analyses of plasma EVs

Total RNA was extracted from plasma EVs and liver tissues using the 3D-Gene RNA extraction reagent (Toray, Kamakura, Japan) and the miRNeasy Mini Kit (Qiagen, Hilden, Germany), respectively, according to the manufacturers’ instructions. The quality of the extracted total RNA was checked using a Bioanalyzer 2100 system (Agilent, Santa Clara, CA, USA). A comprehensive miRNA microarray analysis was performed using 3D-Gene Human miRNA Oligo chips V22 (Toray) designed to detect 2632 miRNAs registered in the miRBase (release 22) database^31–33^. To normalize the signals, three preselected internal control miRNAs (miR-149-3p, miR-2861, and miR-4463) were used for EVs^34^, whereas the global median normalization method was used and the median distribution was set at 25 for liver tissues.

### Serum cytokine and chemokine arrays

Peripheral venous blood obtained before PRI-724 administration was collected in a coagulation tube and centrifuged at 3500 rpm for 20 min at 4 °C. The serum supernatant was stored at -80 °C until use. The levels of serum cytokines and chemokines were measured using Bio-Plex Cytokine Assay Kits (Bio-Rad Laboratories, Hercules, CA, USA), according to the manufacturer’s instructions. Specifically, the Bio-Plex Human Cytokine 48-Plex Panel, Chemokine 40-Plex Panel, and MMP 9-Plex Panel were used. The samples were analyzed in a 96-well plate reader using a Bio-Plex Suspension Array System and Bio-Plex Manager software (Bio-Rad).

### Comprehensive mRNA and miRNA sequencing analysis

C-82, the active form of PRI-724, was kindly provided by PRISM BioLab (Yokohama, Japan). The effect of C-82 in hepatocytes was analyzed using THLE-3 cells (CRL-3583, ATCC, USA), cultured in BEBM basal medium (CC-3171, Lonza, Switzerland) with 10% FBS, whereas the effect of C-82 in hepatic stellate cells was analyzed using the LX-2 Human Hepatic Stellate Cell Line (Sigma-Aldrich, Saint Louis, USA), cultured in DMEM with 2% FBS. LX-2 cells were exposed to human TGF-β1 (0.5 ng/ml) (R&D Systems, Minneapolis, USA) for 24 h before use. After a 6-h exposure to 0.5 μM C-82, total RNA was extracted using the miRNeasy Mini Kit (Qiagen).

### Next-generation sequencing libraries for mRNA and miRNA sequencing

mRNA-seq and miRNA-seq were prepared using the TruSeq Stranded mRNA Sample Preparation kit (Illumina, San Diego, CA, USA) and QIAseq miRNA Library Kit (Qiagen) according to the manufacturer’s instructions, respectively. Optionally, size selection of library for miRNA sequencing was performed using 6% TBE polyacrylamide gel (Invitrogen). Electrophoresis was performed at 120 V for 1 h in Tris–borate-EDTA (TBE) buffer, and the band of interest (173 bp) was eluted using a Spin-X Centrifuge Tube Filter column (Corning Caster).

Each library was quantified by qPCR using the KAPA Library Quantification kit (KAPA). The quantified libraries were mixed at equimolar ratios, and the diluted library pool was loaded onto a NextSeq 1000/2000 P2 reagent v3 (100 Cycles) cartridge with a mixture of 75 pM and 1% phiX, and run on a NextSeq 2000 system (Illumina) in paired-end × 100 nucleotide multiplex and sequenced according to the manufacturer’s instructions. The resulting FASTQ files were analyzed with BaseSpace DRAGEN RNA Pipeline (Illumina) and Gene Globe (Qiagen).

### Detection of miRNAs in liver tissue by in situ hybridization (ISH)

ISH was performed on a Ventana Discovery automated ISH instrument (Ventana Medical Systems, Tucson, AZ, USA) using the RiboMap ISH kit (Ventana). Digoxigenin-labeled, LNA-modified DNA probes targeting miR-887-3p and miR-122, were obtained from Exigon A/S (Vedbaek, Denmark). After deparaffinization of human liver biopsy sections, ISH was performed following the standard protocol described in the manufacturer's RiboMap application note. First, sections were fixed by incubation in formalin-based RiboPrep reagent (Ventana) at 37 °C for 32 min. Acid treatment was then performed using hydrochloride-based RiboClear reagent (Ventana) at 37 °C for 12 min. After cell conditioning by treatment with CC2 reagent (Ventana Medical Systems) for 8 min at 90 °C, the cells were treated with ready-to-use protease 2 reagent. Following an initial denaturing prehybridization step at 91 °C for 8 min, sections were hybridized with an antisense LNA riboprobe (5 pmol/slide) using RiboHybe hybridization buffer (Ventana) for 10 h at 51 °C. Subsequently, three stringency washes were performed using 2 × RiboWash (Ventana) at 51 °C for 3 min. Post-probe fixation was performed using RiboFix reagent for 20 min at 37 °C, and then the tissue sections were incubated with antidigoxigenin alkaline phosphatase Fab fragment 1:800 (Roche Diagnostics, Mannheim, Germany) at 37 °C for 30 min. Signals were detected for 4 h at 37 °C using the BlueMap NBT/BCIP substrate kit. Finally, sections were counterstained with Kernechtrot (nuclear fast red) and covered with glass coverslips.

### Transfection of miRNA mimics

Human hepatic stellate cells (HHSteC) isolated from human liver were purchased from ScienCell Research Laboratories (Carlsbad, CA, USA) and cultured in stellate cell medium (SteCM) (ScienCell Research Laboratories). HHSteC were harvested using TrypLE Express (Life Technologies, Carlsbad, CA, USA) and then reseeded into a collagen I-coated 6-well plate at a density of 1 × 10^5^ cells/well. The cells were then incubated overnight and the culture medium was replaced by SteCM with or without 10 ng/ml TGF-β1 (AF-100-21C; PeproTech, Rocky Hill, NJ, USA). After 24 h, the cells were transfected with a miR-887-3p mimic (mirVana miRNA mimic, Thermo Fisher Scientific, Waltham, MA, USA) or Negative Control #1 (Thermo) using jetPRIME transfection reagent (Polyplus, Illkirch, France), according to the manufacturer’s instructions. Total RNA was extracted 48 h after transfection. The same experiment was also conducted using LX-2 cells, SV40-immortalized human hepatic stellate cells, after their exposure to 5 ng/ml TGF-β1.

### Quantitative reverse transcription-polymerase chain reaction (qRT-PCR)

Total RNA was extracted using the miRNeasy Mini Kit (Qiagen) and reverse transcription was performed using the High-Capacity cDNA Reverse Transcription Kit (Thermo), according to the manufacturers’ instructions. Quantitative PCR was performed using PowerUp SYBR Green Master Mix (Thermo), according to the manufacturer’s instructions. Expression levels of target genes were standardized against the level of *β-actin* in each sample. Primer sequences are shown in Supplemental Table 2.

### Identification of EV-miRNAs of interest

Highly expressed EV-miRNAs with normalized signal intensity > 2^6^ in more than 50% of plasma samples were first selected. Since the measurements were collected in two separate sessions, the bias between the experimental batches was removed using Partek Genomics Suite 7.19.1125 (Supplemental Fig. 6). A cross-validation score, which indicates the robustness of discrimination performance, was calculated for each of the selected miRNAs based on Fisher’s linear discriminant analysis with leave-one-out cross-validation^34^. The miRNAs with area under the receiver operating characteristic (ROC) curve (AUC) values  > 0.8 and cross-validation scores  > 0.75 were selected as having excellent discrimination. The cut-off values that maximized sensitivity and specificity (Youden Index) were set using the ROC curve. Fisher’s linear discriminant analysis was performed using R version 4.1.1 (R Foundation for Statistical Computing, http://www.R-project.org), compute.es package version 0.2–5, hash package version 2.2.6.2, pROC package version 1.18.0, and MASS package version 7.3-58.1.

### Data analysis using public databases

The associations between overall survival and expression levels of HCC tissue mRNAs were assessed using Kaplan–Meier Plotter (https://kmplot.com/analysis/index.php?p=service&cancer=liver_rnaseq)^20^. The associations between overall survival and expression levels of HCC tissue miRNA were assessed using CancerMIRNome (http://bioinfo.jialab-ucr.org/CancerMIRNome/)^35^ based on the TCGA-LIHC dataset^14^. Comparisons of mRNA expression between cell types were performed using Expression Atlas (https://www.ebi.ac.uk/gxa/)^19^. Transcriptome data of liver tissue in patients with liver fibrosis were analyzed based on a previous report^17^.

### Statistical analysis

Unpaired or paired *t*-tests were used to evaluate differences in the distribution of continuous variables between two groups. One-way analyses of variance (ANOVA) were used to evaluate differences in the distribution of continuous variables between more than two groups. χ^2^ tests were used to evaluate differences in the ratios of categorical variables. Correlations between variables were analyzed using Pearson's correlation test. Log-rank tests were used to evaluate differences of survival between two groups. A linear mixed-effects model was used to investigate the effects of responders or non-responders, and PRI-724 administration on plasma EV-miRNA levels and *P*-values for interactions were calculated. A two-sided *P*-value < 0.05 was considered statistically significant. All statistical analyses were performed with R version 4.1.1 (R Foundation for Statistical Computing, http://www.R-project.org), corrplot package version 0.92, and IBM SPSS Statistics version 28 (IBM Corp., Armonk, NY, USA).

### Ethics statement

Written informed consent was obtained from all patients. The PRI-724-2101 trial was approved by the Institutional Review Board of Komagome Hospital (approval number 18–004) and was registered at ClinicalTrials.gov (NCT 03620474). The accompanying miRNA-biomarker study was approved by the Institutional Review Board of Komagome Hospital (approval number 2676) and by the ethics committee of Keio University Faculty of Pharmacy (approval number 220310-8), and was performed in compliance with the Declaration of Helsinki.

### Supplementary Information

Supplementary Information.

## Data Availability

miRNA microarray data have been deposited in the Gene Expression Omnibus database (https://www.ncbi.nlm.nih.gov/geo/) (GSE250179 and GSE250272). RNA-seq data have been deposited in the BioProject database (https://www.ncbi.nlm.nih.gov/bioproject) (PRJNA1059612).
