# Supplementary material for: Plasma extracellular vesicle microRNAs reflecting the therapeutic effect of the CBP/β-catenin inhibitor PRI-724 in patients with liver cirrhosis
Source: Sci Rep. 2024 Mar 15;14:6266. doi: 10.1038/s41598-024-56942-1 (PMC10943077; doi:10.1038/s41598-024-56942-1)
Supplement: Supplementary file 1 — Supplementary Information. [file 41598_2024_56942_MOESM1_ESM.pdf]

Supplementary Materials for

Plasma extracellular vesicle microRNAs reflecting the therapeutic effect of the CBP/β-catenin inhibitor PRI-724 in patients with liver cirrhosis

Mayu Yoshida, Juntaro Matsuzaki*, Koji Fujita, Masamichi Kimura, Tomohiro Umezu, Noi Tokuda, Tomoko Yamaguchi, Masahiko Kuroda, Takahiro Ochiya, Yoshimasa Saito, Kiminori Kimura

*Correspondence to: juntaro.matsuzaki@keio.jp

Supplementary Figures

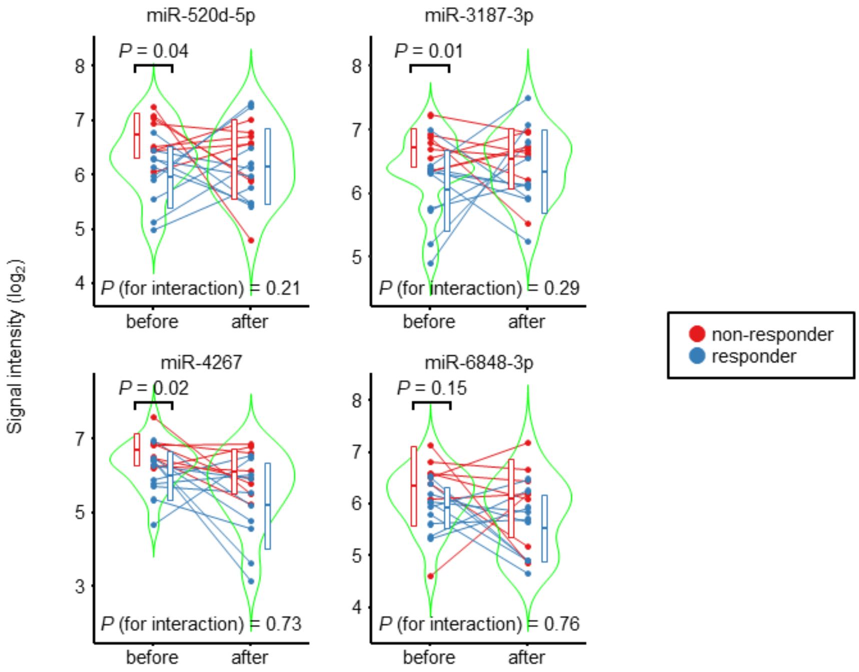

Supplemental Figure 1. Violin plots of the signal intensities of miR-520d-5p, miR-3187-3p, miR-4267, and miR-6848-3p in plasma EVs from responders and non-responders before and after PRI-724 administration. Unpaired *t*-tests were used to analyze statistically significant differences between the responders and non-responders before PRI-724 administration. For each miRNA, the statistical significance of the interaction was calculated to determine whether the effect of PRI-724 on the miRNA level differed between responders and non-responders.

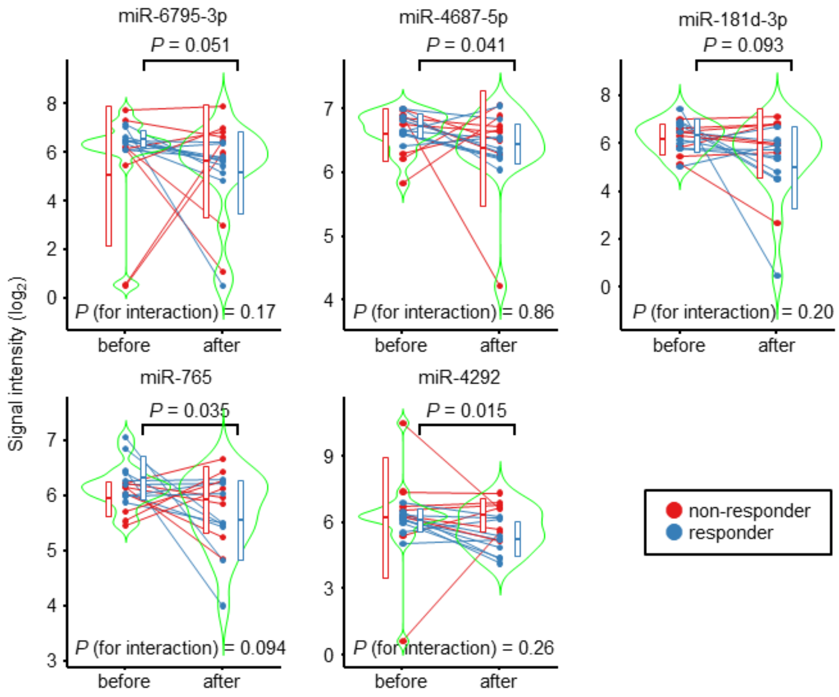

Supplemental Figure 2. Violin plots of the signal intensities of miR-6795-3p, miR-4687-5p, miR-181d-3p, miR-765, and miR-4292 in plasma EVs from responders and non-responders before and after PRI-724 administration. Paired *t*-tests were used to analyze statistically significant differences between the baseline and after PRI-724 administration for the responders. For each miRNA, the statistical significance of the interaction was calculated to determine whether the effect of PRI-724 on the miRNA level differs between responders and non-responders.

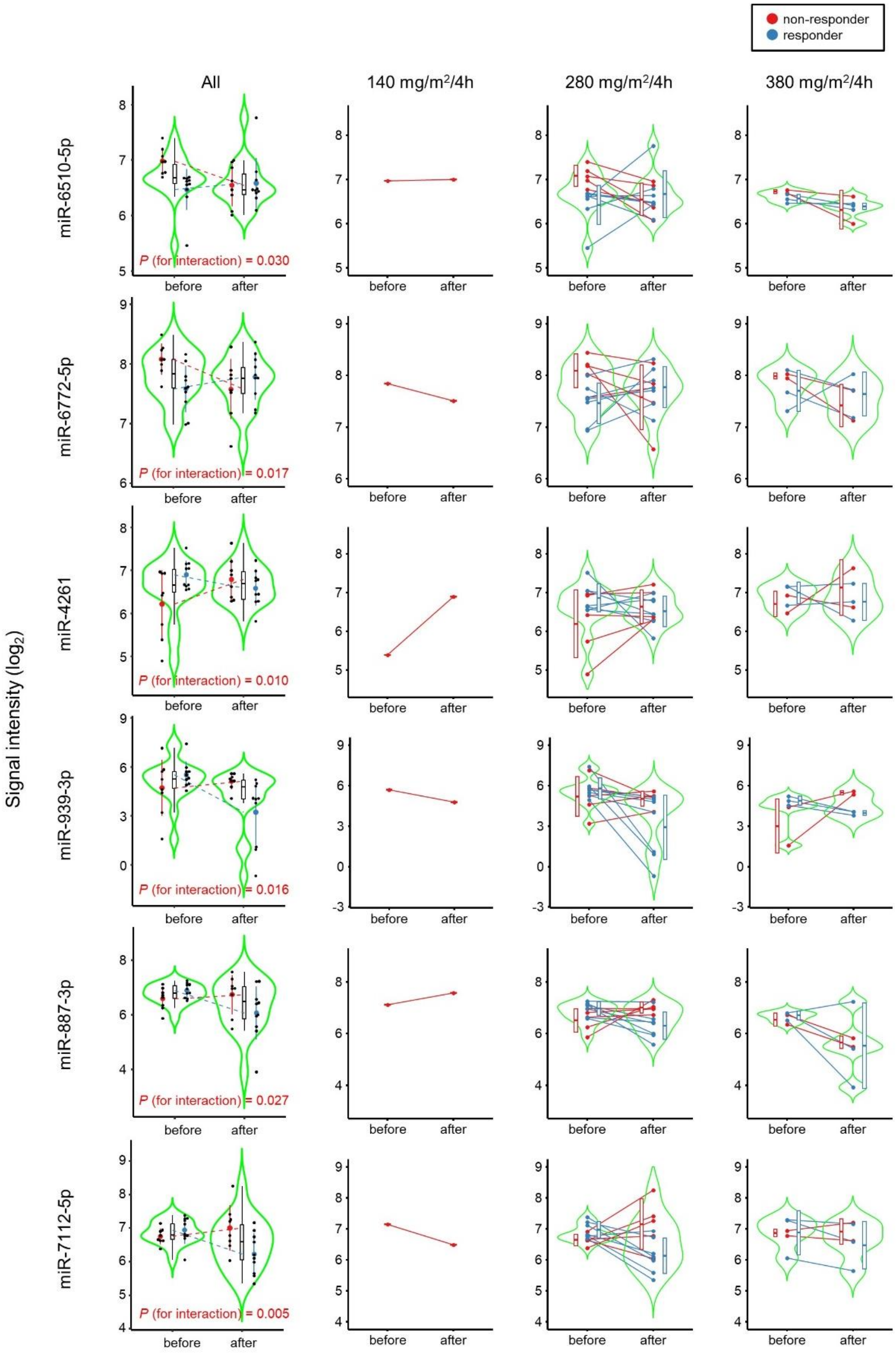

Supplemental Figure 3. Violin plots of the signal intensities of miR-6795-3p, miR-4687-5p, miR-181d-3p, miR-765, and miR-4292 in plasma EVs from responders and non-responders before and after the administration of each dose of PRI-724.

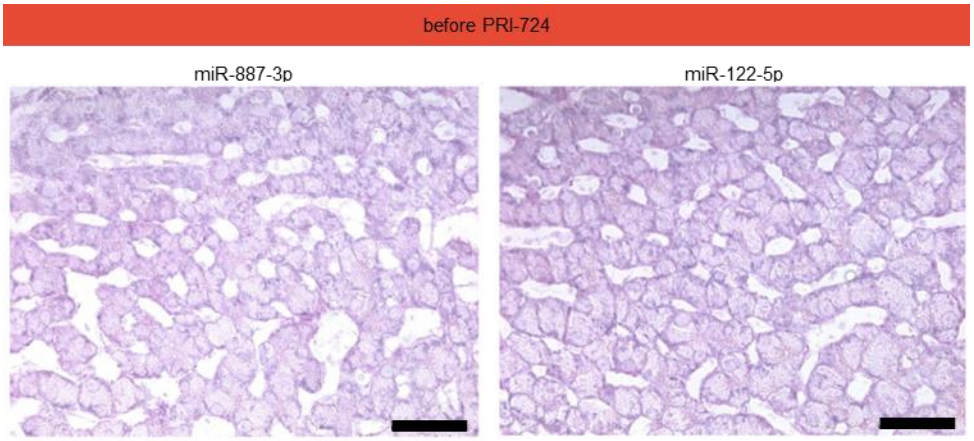

Supplemental Figure 4. In situ hybridization (ISH) of miR-887-3p in liver tissues. Representative staining of miR-887-3p and miR-122-5p is shown. Liver tissues were obtained from patients with liver fibrosis before the administration of PRI-724. miR-887-3p was localized to hepatocytes, as observed previously for the hepatocyte-specific miRNA, miR-122-3p. Scale bar, 50 μm.

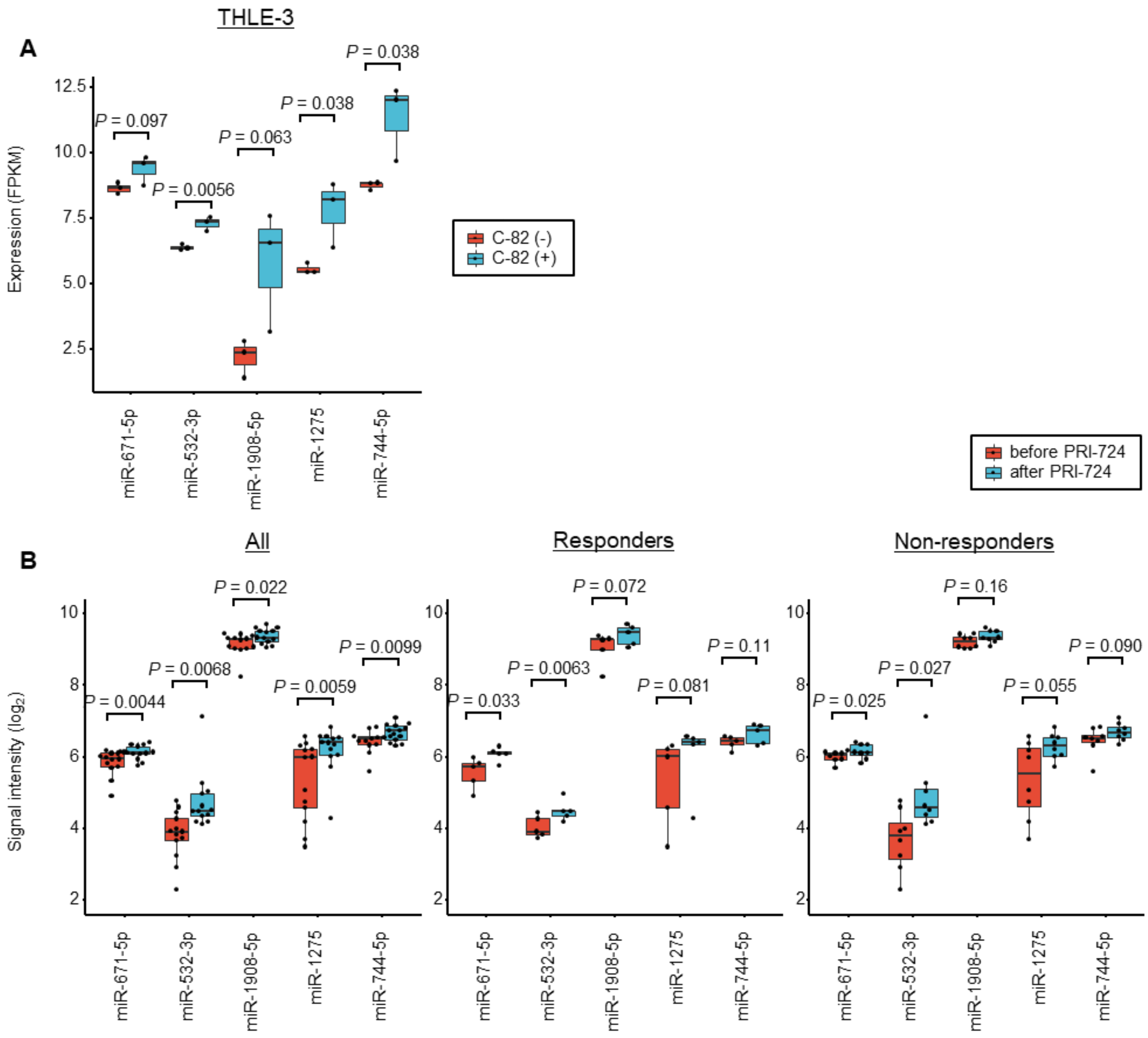

Supplemental Figure 5. miRNAs whose expression levels changed after exposure to C-82.

A) The expression of miRNAs in immortalized hepatocyte cells (THLE-3) with and without exposure to C-82, the active form of PRI-724. Experiments were repeated three times. *P* values were calculated by unpaired *t*-tests.

B) Signal intensities in liver tissue of the miRNAs that were upregulated after exposure of THLE-3 cells (n = 13) to C-82. *P* values were calculated by paired *t*-tests.

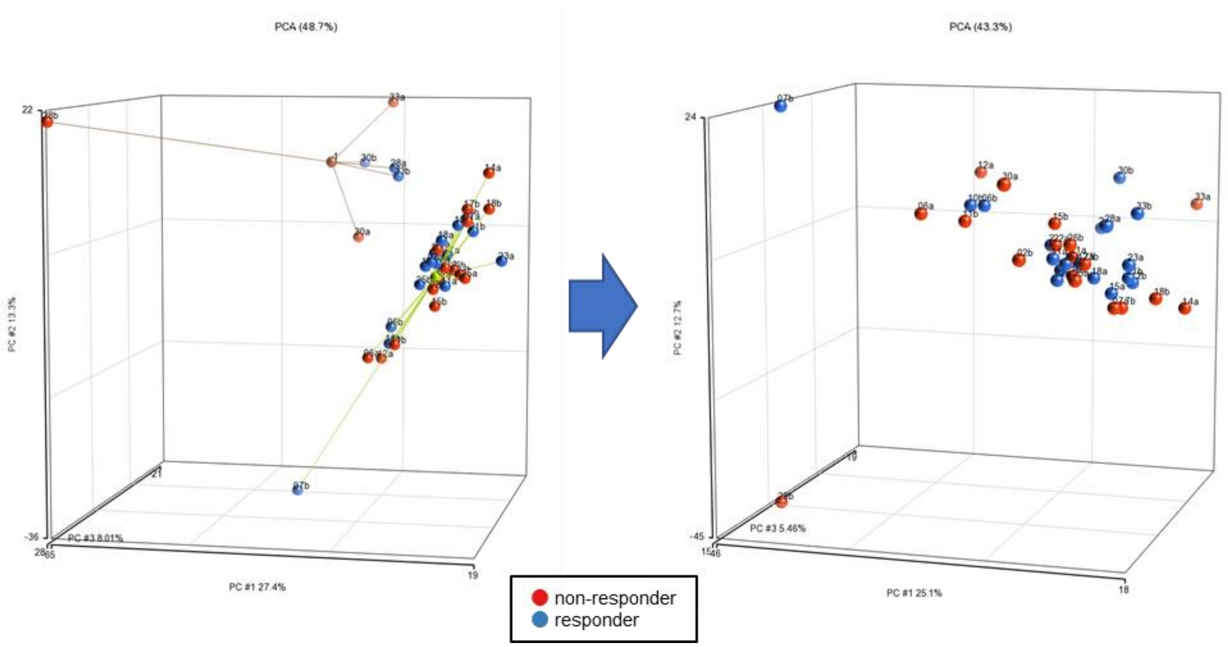

Supplemental Figure 6. Elimination of differences between experimental batches.

Supplemental Table 1. Cytokines expression before treatment

Average (range)	Responder (n = 14)	Non-responder (n = 9)	P-value
MMP-7	6.74 (2.53–19.83)	6.74 (2.45–11.53)	1.00
MMP-8	8.8 (4.29–19.61)	6.98 (5.08–12.32)	0.21
b-NGF	1.27 (ND–13.9)	0.55 (ND–3.03)	0.52
CTACK	1139.11 (717.07–1547.66)	1081.48 (449.05–1855.47)	0.70
Eotaxin	123.04 (51.39–201.61)	129.56 (51.64–238.34)	0.78
FGF basic	30.89 (23.13–40.04)	32.04 (23.13–40.04)	0.56
G-CSF	123.42 (52.26–190.16)	118.20 (74.36–167.21)	0.73
GM-CSF	1.51 (ND–4.26)	2.74 (ND–14.45)	0.44
GRO-a	141.00 (ND–675.45)	146.59 (ND–546.16)	0.95
HGF	966.34 (322.04–2390.77)	650.02 (407.97–1036.03)	0.06
IFN-a2	4.49 (0.51–8.02)	4.24 (ND–7)	0.81
IFN-g	18.39 (10.28–31.7)	17.62 (11.92–22.61)	0.74
IL-1a	14.97 (7.76–24)	12.57 (7.76–18.58)	0.18
IL-1b	2.39 (1.13–3.26)	2.49 (2.2–2.97)	0.58
IL-1ra	309.25 (143.02–610.83)	302.05 (121.12–480.53)	0.89
IL-2	0.85 (ND–5.88)	0.69 (ND–1.68)	0.78
IL-2Ra	69.99 (25.12–107.87)	57.72 (34.15–86.75)	0.18
IL-3	ND	ND	ND
IL-4	5.89 (3.2–8.14)	5.82 (3.81–8.64)	0.93
IL-5	8.8 (ND–123.17)	14.86 (ND–74.41)	0.63
IL-6	15.3 (ND–167.58)	6.04 (ND–41.7)	0.47
IL-7	14.24 (1.58–22.63)	16.79 (8.04–33.12)	0.40
IL-8	17.05 (2.62–40.51)	14.68 (2.97–26.92)	0.56
IL-9	235.78 (155.59–308.34)	249.62 (211.43–282.03)	0.32
IL-10	3.35 (ND–8.39)	3.03 (0.41–4.66)	0.69
IL-12 (p70)	2.38 (ND–9.39)	1.69 (ND–5.63)	0.55
IL-12 (p40)	41.8 (9.08–92.94)	30.74 (14.43–66.59)	0.21
IL-13	4.06 (1.16–14.19)	6.79 (1.46–22.89)	0.28
IL-15	ND	ND	ND
IL-16	65.07 (29.87–99.14)	58.34 (33.12–83.03)	0.42
IL-17A	11.45 (7.17–19.97)	10 (7.44–13.49)	0.21
IL-18	42.06 (9.96–135.39)	33.22 (11.12–84.01)	0.43
IP-10	807.04 (268.28–1987.84)	818.82 (529.38–1700.75)	0.95
LIF	30.65 (13.43–72.51)	26.44 (16.12–33.03)	0.34
M-CSF	33.5 (10.77–70.78)	25.48 (18.38–48.68)	0.19
MCP-1 (MCAF)	49.05 (14.53–94.41)	64.25 (27.65–110.72)	0.18
MCP-3	0.99 (ND–2.21)	1.07 (ND–2.21)	0.84
MIF	486.44 (228.52–981.7)	425.47 (183.49–620.92)	0.46
MIG	346.75 (193.82–767.77)	426.75 (167.04–914.95)	0.39
MIP-1a	3.45 (1.74–6.58)	3.01 (1.85–5.29)	0.39
MIP-1b	145.78 (105.86–193.81)	147.76 (123.7–178.57)	0.82
PDGF-bb	2349.26 (1038.58–5793.81)	2593.92 (1366.92–3951.96)	0.61
RANTES	4889.33 (1224.44–15077.7)	6205.32 (2207.08–12498.62)	0.38
SCF	134.22 (76.68–306.12)	111.64 (51.13–186.49)	0.31
SCGF-b	142944.14 (67847.25–210069.12)	148566.21 (75418.97–182867.69)	0.73
SDF-1a	1667.09 (1153.31–2505.92)	1573.9 (1122.94–2359.48)	0.61
TNF-a	128.41 (25.53–1275.91)	38.42 (19.71–61.68)	0.33
TNF-b	738.14 (515.42–1034.25)	771.01 (674.08–903.75)	0.52
TRAIL	29.97 (17.65–47.27)	32.8 (22.68–40.93)	0.39
VEGF	105.87 (ND–1411.76)	10.68 (ND–96.11)	0.36

ND, not determined

Supplemental Table 2. Primer sequences

Gene	Forward	Reverse
αSMA	CTGTTCCAGCCATCCTTCAT	GGCAATGCCAGGGTACATAG
GPR173	GGTAACGCCATCTTGTCCCT	AGCTGAGTGCACTGAAGGTC
β-actin	ACTCTTCCAGCCTTCCTTCC	AGCACTGTGTTGGCGTACAG
